# Guidelines for the diagnosis and treatment of follicular lymphoma in China

**DOI:** 10.7497/j.issn.2095-3941.2013.01.006

**Published:** 2013-03

Follicular lymphoma (FL) is a common subtype of B-cell lymphoma. Based on the updated National Comprehensive Cancer Network guidelines and related medical data, the authors formulated the FL guidelines for China by combining the diagnostic level and current situation of lymphoma.

## Definition

Follicular lymphoma (FL) is a common type of non-Hodgkin’s lymphoma (NHL), accounting for 22% to 35% of NHL in western countries. The proportion in China, 8.1% to 23.5%, is less than that in western countries, although its incidence in China has increased with time. Compared with foreign countries, the age of onset is younger in China, and in terms of geographical distribution, the incidences in coastal regions and economically developed areas are higher^1^^^-^^^3^.

FL is from germinal-center B-cells, and is manifested morphologically as a tumor retaining follicular growth. FL comprises a group of malignant lymphoproliferative diseases including follicular center cells (small cleaved cells) and follicular center matricytes (large non-cleaved cells). Under microscopy, FL sometimes appears to be associated with diffused components. Based on different follicular components and their proportions, FL can be divided into the following (1) follicle-dominant type (proportion of follicles >75%); (2) follicle and diffuse mixed type (follicles accounting for 25% to 75%); and (3) focal follicle (proportion of follicles <25%)^4^^,^^5^.

## Diagnosis, staging, prognosis, and differential diagnosis

### Diagnosis

The diagnosis of FL is mainly based on histopathological examination, including immunohistochemical and morphological examination, although it can also be based on the results of flow cytometric and cytogenetic analyses, if necessary.

According to the lymphoma classification of the World Health Organization, FL can be further divided into stages 1 to 3. In Stage 1, 0 to 5 matricytes are present per high-power field; in Stage 2, 6 to 15 matricytes are present per high-power field; and in Stage 3, the number of matricytes per high-power field >15, among which the remaining few centrocytes are referred to as Class 3A, while Class 3B is characterized by infiltrated flaky central matricytes and the absence of centrocytes. The clinical manifestation of FL in Class 1-2 and 3A is inertia, and that in Class 3B is manifested by invasion similar to diffuse large B-cell lymphoma (DLBCL). Currently, FL in Class 1-2 is based on the treatment principles of indolent lymphoma. FL in Classes 3A and 3B is treated with the treatment principles of DLBCL, and the clinical effects are similar. After a few years of treatment, the diagnosis of several FL patients changes to invasive lymphoma, mainly invasive DLBCL with poor prognosis^6^^,^^7^.

FL has a characteristic immunophenotype and B-cell markers are expressed in cell surface. Immunohistochemical detection generally uses the following group of indices: CD20, CD3, CD5, CD10, Bcl-6, Bcl-2, CD21, CD23, and cyclinD1. Ki-67 is typically recommended for assessment. The typical immunohistochemical markers are CD20+, CD23+/-, CD10+, CD43-, Bcl-2+, Bcl-6+, CD5-, and CCND1-, while Bcl-2- or CD10- also appear in several cases. Molecular genetic testing can help identify bcl-2 rearrangement, while cytogenetics or fluorescence in situ hybridization inspection t (14;18) or t (8;14) can facilitate diagnosis.

### Inspection, staging, and prognosis of FL

The diagnostic test of FL is similar to other indolent lymphoma inspections. The necessary steps include the following: general physical examination, particularly to check if superficial lymph node, liver, and spleen are enlarged; laboratory examination, including complete blood cell inspection, blood biochemical inspection, as well as serum lactate dehydrogenase (LDH) level, hepatitis B and C, and HIV-related detection; and imaging examinations via enhanced computed tomography (CT) detection of neck, chest, abdomen, and pelvic cavity and via bilateral or unilateral bone marrow biopsy plus smear examination, which are recommended to be performed routinely. The sample for bone marrow biopsy should be at least larger than 1.6 cm. Using positron emission tomography (PET)/CT is helpful in detecting some non-palpable lesions, but its clinical value is lower than those found in DLBCL and Hodgkin's lymphoma subtype. In addition, PET/CT can help diagnose FL transformation.

To predict FL prognosis, the standard of FL International Prognosis Index (FLIPI) is as follows: for FLIPI-1, age ≥60, Ann Arbor staging includes stages III to IV, hemoglobin (HBG) <120 g/L, serum LDH >upper limit of normal range, and involved lymph nodes ≥5. Each indicator refers to a score of 1. According to the scores, FL patients can be divided into three risk groups, namely, low risk, intermediate risk, and high risk. In recent years, with the increasingly common use of IDEC-C2B8 (rituximab) for treating FL, the new clinical prognostic scoring system, FLIPI-2, is considered an improved version of FLIPI-1. FLIPI-2 includes the following factors: β_2_-microglobulin > upper limit of normal range, maximal lymph node diameter>6 cm, bone marrow being violated, HGB <120 g/L, and age >60 (**Table 1**).

**Table 1:** FLIPI-1 and FLIPI-2

Characteristics	Poor factors	
	FLIPI-1	FLIPI-2
Lymph node	>4 areas of lymph node	Maximal diameter of lymph node >6 cm
Age, years	>60	>60
Serum marker	Increased LDH	Increased β_2_-microglobulin
Staging	Late stage (Ann Arbor III–IV)	Bone marrow violated
Hemoglobin	<12 g/dL	<12 g/dL

Low risk, 0 to 1; Intermediate risk, 2; High risk, 3 to 5.

## Treatment of FL

### Treatment indicators

Localized radiation therapy is mainly adopted for FL patients in Stages I and II. The disease-free survival is extended for most patients. Therefore, radiation therapy or radiation therapy associated with general immunochemotherapy should be adopted as soon as possible.

Currently, FL patients in Stage II with abdominal mass and Stages III to cannot be cured with the available treatment methods. Most patients have slow disease progression and can maintain better quality of life, even if treatment is not for a long time. Thus, a patient can be treated if one of the following treatment indicators is exhibited (**Table 2**).

**Table 2:** Treatment indicators

B symptoms	>38 °C, fever of undetermined origin, night sweating, weight loss without reason >10% within 6 months
Abnormal signs	Enlarged liver and spleen; pleural effusion; ascitic fluid
Damage to vital organs	Accumulated diseases causing organ dysfunction
Blood index	Hypocytosis (hematocyte <1.0×10^9^/L and/or platelet <100×10^9^/L);
	Leukemia performance (malignant cell >5.0×10^9^/L)
	LDH higher than normal value (Hb <120 g/L)
	β_2_-microglobulin ≥3 mg/L
Giant mass	Diameters of three tumors ≥5 cm or diameter of one tumor ≥7 cm (patients in Stages III–IV)
Continuous tumor progression	Tumor enlarged about 20% to 30% within 2 to 3 months, and enlarged about 50% within 6 months
Meet the inclusion criteria of the clinical experiment	To confirm according to the detailed requirements of the clinical experiment

Evaluation before treatment.

The following items should be detected before treatment: (1) disease history; (2) physical examination aimed at identifying possible areas of lymph node accumulation and the sizes of the cerebral arterial circle and liver and spleen; (3) physical condition; (4) B symptoms; (5) complete blood count and biochemical routine; (6) CT scans of the neck, chest, abdomen, and pelvis; (7) hepatitis B virus detection; (8) bone marrow biopsy and smear; (9) routine electrocardiogram.

Ultrasonic cardiogram of the left ventricular ejection fraction, PET/CT, β_2_-microglobulin, uric acid, serum protein electrophoresis, and/or quantitative immunoglobulins and Hepatitis C-related examination can also be performed, if necessary.

### Front-line treatment options of FL patients in Stages I to II

FL patients in level 3 should be treated based on DLBCL treatment strategy. Enough clinical evidence has shown that involved field radiation therapy (IFRT) is an ideal treatment option for FL patients in stages I to II of levels 1 and 2. Improved long-term survival rate can be achieved by simple radiation treatment, with a radiation dose of 30 Gy to 36 Gy. Although the efficacy of general immunochemotherapy associated with the radiation treatment for FL patients in Stages I to II has yet to be fully explored, several reports have pointed out that such treatment could improve survival. Watchful monitoring is recommended if the risk of adverse reactions of FL patients for IFRT is estimated to be greater than the probability of clinical benefits. Front-line-associated immunochemotherapy may be recommended for patients in Stages I to II with high tumor burden or patients in FLIPI intermediate and high risk (>1 score)^8^.

### Front-line treatment of FL patients in Stages III to IV

Compared with FL in Stages I to II, FL in Stages III to IV is generally considered an incurable disease. The watchful monitoring strategy can be adopted if patients have no treatment indicators (**Table 2**). For FL patients in Stages III to IV with treatment indicators, the treatment options are more current, and the general principle is to choose a highly individual treatment program based on age, general physical conditions, complications, and treatment goal.

Immunochemistry is currently the most commonly selected treatment mode at home and abroad. The treatment program with 8 courses of rituximab associated with chemotherapy has become the preferred standard program at home and abroad for the initial treatment of FL patients. Regardless of which treatment is chosen (i.e., CHOP, CVP program, or fludarabine associated with rituximab), all treatment methods have been shown to improve recent and long-term effects significantly, including overall survival. Therefore, routine dose of associated chemotherapy plus rituximab is suggested for relatively young patients with better constitution^9^^,^^10^.

Meanwhile, researchers have still not reached a consensus on the optimal front-line treatment program for late-stage FL patients, either by chemotherapy associated with rituximab or single-agent rituximab. Nevertheless, the result of a recent FOLL05 experiment shows that R-CHOP program is better than R-CVP or R-FM program based on risks and benefits. Several studies have shown that fludarabine has toxicity on bone marrow stem cells and is related to secondary tumor. Therefore, premature use should be avoided, particularly for patients with autologous hematopoietic stem cell transplantation (ASCT).

For weak and old patients who are unable to endure combination chemotherapy, single-agent rituximab, single-agent chemotherapy, or rituximab associated with single-agent chemotherapy can be selected for front-line treatment. In addition, supportive treatment should be enhanced.

### The treatment rule of relapsed FL patients

Regardless of the type of induced immunochemotherapy adopted, relapse can occur after a period of disease remission. As of this writing, the standard treatments for relapsed, intractable FL patients have yet to be unified. The option for salvage therapy depends on the curative effects of previous programs, release time, age of patients, physical conditions, pathologic types at relapse, and treatment goal. For the relapsed patients without transformation and with long-term remission after front-line treatment, the original program or other front-line programs can be used. For the early relapsed patients (<12 months), non-cross drug-resistant program (e.g. fludarabine, can be used as salvage program if relapse occurs after CHOP treatment) can be selected and applied. The effective rate of rituximab for treating relapsed FL is around 45%, whereas the rate for CR is 6%. Rituximab can also increase the effect of salvage chemotherapy. The salvage chemotherapy options include CHOP, fludarabine-based program, CVP, and radioimmunotherapy. New drugs and new combined programs can also be considered. Thus, it is recommended that ASCT be adopted in the treatment of relapsed young patients^9^^,^^10^.

### Maintenance treatment of FL

FL patients with a long disease history and slow progress are more sensitive to various treatments. Thus, maintenance treatment is suitable for these patients after remission induction. Numerous clinical studies and meta-analysis results have proven that, for FL patients after front-line treatment or one more remission induction of relapse, the maintenance treatment via single-agent rituximab improves long-term survival^11^^^-^^^14^. Therefore, the recommended treatment for patients being initially treated or relapsed patients after induction chemotherapy and complete remission (CR) or partial remission (PR) is one maintenance treatment by single-agent rituximab every 2 to 3 months, for a total of 2 years. However, the probability of infection can increase after maintenance treatment. Close follow-up and observation should be given to hepatitis B patients^15^.

### Treatment of conversion FL lymphoma

Around 20% to 70% of FL that occur in patients can be clinically transformed into other more invasive lymphomas. The most common of these invasive lymphomas is DLBCL, which has an annual incidence rate of 2% to 3% and has continued to increase for at least 15 years. After this period, the transformation risk decreases gradually, after which the transformation is no longer affected regardless of whether or not FL patients have ever been treated. Most patients, after undergoing transformation, have poor prognosis, with a median survival time of 10 to  18 months. Uneven values in FDG-PET scanning and increasing standardized uptake value can show the transformation, which should be verified by biopsy.

Currently, no standard therapeutic measure exists for transformative FL; thus, the therapeutic measure of transformed invasive lymphoma can be adopted. The patients treated by mild chemotherapy or those who have not recieved chemotherapy can choose anthracycline-based combined chemotherapy ± radiation treatment or chemotherapy ± rituximab, in order to achieve better outcome. If patients have been previously treated strongly by numerous kinds of chemotherapy programs, IFRT or other chemotherapy programs can be considered. These patients with poor prognosis are recommended to participate in clinical trials. If patients are sensitive to chemotherapy, hematopoietic stem cell transplantation, particularly ASCT, should be considered for administration after they are eased again into treatment. Meanwhile, allogeneic hematopoietic stem cell transplantation (allo-HSCT) may be attempted in a small number of highly selected patients.

### Hematopoietic stem cell transplantation

The therapeutic action of high-dose chemotherapy supported by ASCT on FL patients in Stages III to IV remains controversial. Various studies have shown that ASCT is not highly effective for patients being eased initially, and that autologous transplantation can extend the survival time of FL patients with sensitive relapse (1 to 4 relapses by preference). Therefore, FL patients in Stages III to IV who are still sensitive to chemotherapy after numerous relapses are encouraged to participate in such kind of clinical trial, especially if they are young or have good physical conditions with improved function of vital organs. With the continuous progress of allo-HSCT, myeloablative or non-myeloablative allo-HSCT have initially shown long-term survival benefits for several patients. However, the problem of high transplantation-related mortality rate still needs to be solved. Currently, allo-HSCT is only suitable for a small number of patients for research.

## Treatment of untoward effects

Details are found in the relative treatment guidelines of DLBCL in China.

## Criterion of therapeutic effects

Currently, the 1999 guidebook published by the International Working Group and the 2007 guidebook revised by the International Coordination Scheme are adopted as the criteria of the therapeutic effects of lymphoma. The criterion in the 1999 version is based on the reduced size of the swelled lymphadenopathy measured by CT, and the level of bone marrow being infiltrated confirmed by smear and biopsy. The therapeutic effects are divided into CR, uncertain complete remission (CRu), PR, steady (SD), and relapse or development (PD). FDG-PET/CT is added into the criterion in the 2007 version. PET/CT can determine if the residual mass is PR or CR; thus, the revised criterion cancelled CRu, and kept CR, PR, SD, and PD. Imaging review should be done at least 3 weeks after the end of chemotherapy. The detailed evaluation of therapeutic effects is shown in **Tables 3**** and ****4**.

**Table 3:** Criteria of therapeutic effects (excluding PET)

Classification	Physical examination	Lymph node	Extranodal mass	Bone marrow
CR	Normal	Normal	Normal	Normal
CRu	Normal	Normal	Normal	Unsure
	Normal	Normal	Reduce >75%	Normal or unsure
PR	Normal	Normal	Normal	Positive
	Normal	Reduce ≥50%	Reduce ≥50%	Unrelated
	Liver/spleen reduced	Reduce ≥50%	Reduce ≥50%	Unrelated
PD	Swelled liver/spleen and new lesion	New lesion or raw lesion enlarged	New lesion or raw lesion enlarged	Relapse

**Table 4:** Revised criteria of therapeutic effects (including PET)

Therapeutic effects	Definition	Lymph node enlarged	Liver, spleen	Bone marrow
CR	All lesions disappeared	① Before treatment, FDG shows high affinity or PET is negative; after treatment, any size of lymph node is PET-negative.	Liver and spleen could not be touched; the node disappeared.	Re-biopsy is negative; if morphology cannot make a definite diagnosis, immunohistochemistry should be used, and the result should be negative.
		② Affinity of FDG is uuncertain or PET is negative; CT shows lymph nodes in normal size.		
PR	Measurable lesions become smaller, no new lesion	SPD of six biggest lesions shortened ≥50% without other enlarged lesions.	For all lesions, SPD shortened ≥50% (the biggest transverse diameter of single lesion shortened ≥50%), no liver or spleen enlargement.	If the result is positive before treatment, the cell type should not be used as the evaluation standard of effects; cell types should be clear.
		① Before treatment, FDG shows high affinity or PET is positive; one or many PET-positive lesions are present in the original lesion.		
		② Affinity of FDG is uncertain or PET is negative; CT shows shortened lesions.		
SD	Failure to reach the standard of CR/PR or PD	① Before treatment, FDG shows high affinity or PET is positive; after treatment, PET is still positive in the original lesion, and CT or PET showed no new lesions.		
		② Affinity of FDG is uncertain or PET is negative; CT shows no change in the lymph node size.		
Relapse or PD	Any new lesion; or enlargement of the diameter of the original lesion ≥50%	New lesion with DM >1.5 cm appears; SPD of multi-lesions enlarged ≥50%; the biggest DM of the single lesion with the smallest DM >1 cm before treatment enlarged ≥50%; before treatment, FDG shows high affinity or PET is positive; after treatment, PET is negative.	For any lesion, SPD enlarged >50%	New lesion or relapse

## Follow-up

Follow-up must be conducted every 2 to 3 months in the first year for patients in remission stage (CR or PR) after all treatment methods. Then, one follow-up every 3 months should be conducted in the second year, followed by one follow-up every 6 months. The follow-up sessions may also be scheduled according to clinical indications. Follow-up content covers repeat diagnostic test, imaging examination based on the clinical situations (i.e., depending on disease region and clinical feature), and physical examination.

## **Appendix:** The main treatment programs of FL

### Front-line treatment program

Single agent: Chlorambucil and/or single-agent rituximab. This program is suitable for old and weak patients.

R-CHOP: Rituximab is administered in the first day, repeated every 3 to 4 weeks, 8R-6CHOP. This program is one of the most common standard treatment programs for FL patients. For old patients with cardiac dysfunction, epidoxorubicin (epirubicin), pirarubicin, or liposomal doxorubicin can be used to replace conventional doxorubicin.

R-CVP: This program is also one of the most common standard treatment programs for FL patients. R-CVP is milder than R-CHOP, and is suitable for old patients with cardiac dysfunction.

R-F: Rituximab is administered in the first day, whereas fludarabine is given for 2 to 4 d, and then repeated every 28 d. Note: immunological suppression is more obvious, and patients can easily be infected.

Consolidation or maintenance treatment after front-line treatment: Rituximab is used for maintenance treatment after immunochemotherapy remission. The dosage of rituximab is 375 mg/m^2^, once every 2 to 3 months for a total of 2 years. Notes: patients in CR/CRu/PR after inductive treatment will be given maintenance treatment; low immunoglobulinemia can appear during maintenance treatment, and patients can recover on their own even if rituximab is not administered.

### Second-line treatment program

If a longer non-treatment gap exists after front-line treatment, the original treatment program can be considered to be continuously used once relapse occurs.

R-FC: Rituximab is administered in the first day, whereas fludarabine and CTX are given for 2 d to 4 d, to be repeated every 28 d. Note: preventive *Pneumocystis carinii* pneumonia treatment should be considered.

R-F: Rituximab is administered in the first day, whereas fludarabine is given for 2 d to 4 d, to be repeated every 28 d.

The following DLBCL second-line program can be considered: ESHAP (etoposide + methylprednisolone  + cis-platinum + Ara-C) ±rituximab; GDP (gemcitabine + dexamethasone + cis-platinum) ±rituximab; GemOX (gemcitabine + oxaliplatin) ±rituximab; ICE (ifosfamide  + carboplatin + etoposide) ±rituximab; single-agent Thalidomide; full oral PEPC program. Note: the highly individual dose adjustment and time arrangement should be selected according to the actual conditions of the patients.

### Second-line maintenance treatment program

A dose of 375 mg/m^2^ of rituximab is administered for 2 years, and repeated every 2 to 3 months. Notes: CR/PR patients undergo maintenance treatment after inductive treatment; prognosis is poor for patients with repeated relapse; thus, these patients are encouraged to participate in clinical research.
